# Insight into Bio-metal Interface Formation *in vacuo*: Interplay of S-layer Protein with Copper and Iron

**DOI:** 10.1038/srep08710

**Published:** 2015-03-04

**Authors:** Anna A. Makarova, Elena V. Grachova, Vera S. Neudachina, Lada V. Yashina, Anja Blüher, Serguei L. Molodtsov, Michael Mertig, Hermann Ehrlich, Vera K. Adamchuk, Clemens Laubschat, Denis V. Vyalikh

**Affiliations:** 1 Institut für Festkörperphysik, Technische Universität Dresden, 01062 Dresden, Germany; 2 Department of Physics, St. Petersburg State University, 198504 St. Petersburg, Russia; 3 Department of Chemistry, St. Petersburg State University, 198504 St. Petersburg, Russia; 4 Department of Chemistry, Moscow State University, 119991 Moscow, Russia; 5 Professur für Physikalische Chemie, Mess- und Sensortechnik, Technische Universität Dresden, 01062 Dresden, Germany; 6 Institut für Experimentelle Physik, Technische Universität Bergakademie Freiberg, 09599 Freiberg, Germany; 7 European XFEL GmbH, 22761 Hamburg, Germany; 8 ITMO University, 197101 St. Petersburg, Russia; 9 Kurt-Schwabe-Institut für Messund Sensortechnik e.V. Meinsberg, 04736 Waldheim, Germany

## Abstract

The mechanisms of interaction between inorganic matter and biomolecules, as well as properties of resulting hybrids, are receiving growing interest due to the rapidly developing field of bionanotechnology. The majority of potential applications for metal-biohybrid structures require stability of these systems under vacuum conditions, where their chemistry is elusive, and may differ dramatically from the interaction between biomolecules and metal ions *in vivo*. Here we report for the first time a photoemission and X-ray absorption study of the formation of a hybrid metal-protein system, tracing step-by-step the chemical interactions between the protein and metals (Cu and Fe) *in vacuo*. Our experiments reveal stabilization of the *enol* form of peptide bonds as the result of protein-metal interactions for both metals. The resulting complex with copper appears to be rather stable. In contrast, the system with iron decomposes to form inorganic species like oxide, carbide, nitride, and cyanide.

Novel metal-biohybrids with unusual combinations of chemical and physical properties are attracting increasing attention in the field of bioinspired materials chemistry. One important aspect in this growing field is gaining insight into the fundamental mechanisms of metal-biomolecule interactions in a particular bonding environment. Note that essential progress has been made in understanding these interactions *in vivo*, where they play an important role in functioning of living organisms^1^,^2^. Nevertheless, there remains much work to be done in terms of discovering important chemical bonding information for artificial metal-biohybrid systems, specifically for those synthesized in non-liquid environments. This chemistry may differ dramatically from those interactions observed between biomolecules and metal ions *in vivo*. The rapidly developing sectors of bioelectronics, nanomedicine and biorobotics^3^,^4^,^5^,^6^,^7^ drive investigations into these fundamental processes in such artificial systems. Particular attention should be paid to the synthesis and chemistry of the hybrids under ultra high vacuum (UHV) conditions, since many applications are created or operated only *in vacuo*^8^,^9^,^10^,^11^,^12^,^13^,^14^. This includes scenarios like integrating biomolecules into electronic circuits, biomolecule wiring with metal electrodes, or using them as templates to grow metallic or magnetic nanocluster arrays. Yet, in most cases the metal-biomolecule interactions of these artificial systems synthesized *in vacuo* literally remain *terra incognita*, specifically since for most applications of that sort the metal is deposited in its elemental form, which makes the data obtained so far on the protein-metal ion interaction in the biosystems inapplicable. These applications provide a strong impetus for investigating the chemical interaction between biomolecular objects and elemental transition metals during their deposition in a vacuum.

Here, we report photoemission (PE) and X-ray absorption studies on the interaction between proteins and transition metals deposited in UHV. For our investigations, we used (i) regular bacterial surface layer proteins (S-layers, SL), as well as (ii) metals that are most important for electronic and magnetic applications such as Cu and Fe. The choice of the S-layer protein as a model substrate for the metal/protein interaction studies was motivated by its high stability in a dry form and under vacuum conditions (S-layers are among the most stable protein membranes provided by nature), and the fact that such proteins have been often employed as biomolecular templates for metal deposition and decoration^15^,^16^. It should be underlined that in contrast to multiple previous peptide/metal interaction studies, the metals deposited herein were in their elemental form, which to a great extent defined the chemical reactivity and unusual reaction mechanisms.

## Results and Discussion

We examine the interplay between protein and metal by looking at this process from both sides; *i.e*. we analyze the spectral features of the protein substrate and of the deposited metal. Here, we show the results from coverage-dependent spectral studies.

**Figure 1:**
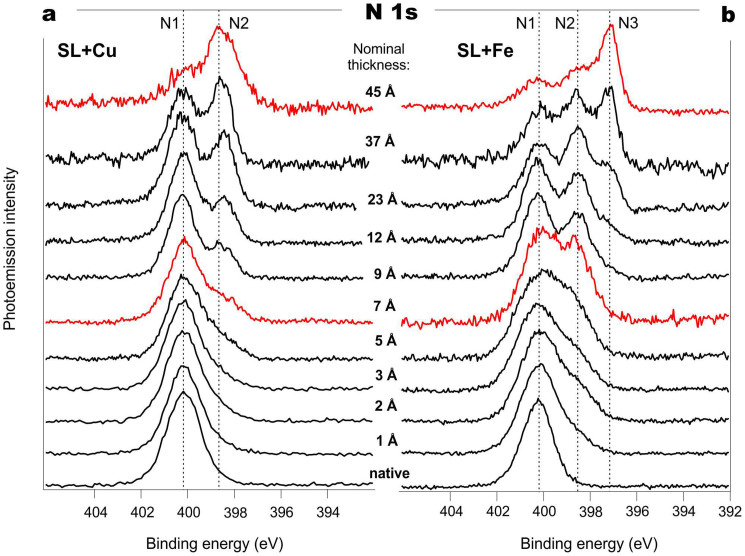
Core-level N 1s spectra taken for the native S-layer and after gradual dose deposition of copper (a) and iron (b) on top. The spectra were normalized each to its maximum intensity.

First, we consider the spectral changes seen in the protein, which were mainly monitored by recording PE N 1s core-level spectra. Figures 1a and 1b show spectra taken during the gradual deposition of Cu and Fe onto the SL samples, which were all freshly prepared and introduced into UHV chambers. As can be seen, the N 1s spectrum of the native SL can be described by one broad component positioned at 400.1 eV (*N1* feature)^17^,^18^,^19^, with unresolved fine structure. This structure is expected as about 81% of the nitrogen atoms are in peptide bonds, nearly 8% participate in formation of similar amide bonds, and about 10% belong to amine groups in side chains. The remaining minor fraction is due to nitrogen in indole (trp) and imidazol (his)^20^.

Upon gradual deposition of either Cu or Fe, the N 1s spectra reveal notable changes even at low coverage (below 7 Å, Figure 1a). An additional spectral feature *N2* appears, shifted by 1.7 eV towards lower binding energies (BEs) relative to the main peak position. The corresponding BE for *N2* is 398.4 eV, which is a sign of the double C = N bond^21^,^22^,^23^,^24^. Note here that for this “low coverage” stage, no visible changes in O 1s and C 1s spectra have been detected.

**Figure 2:**
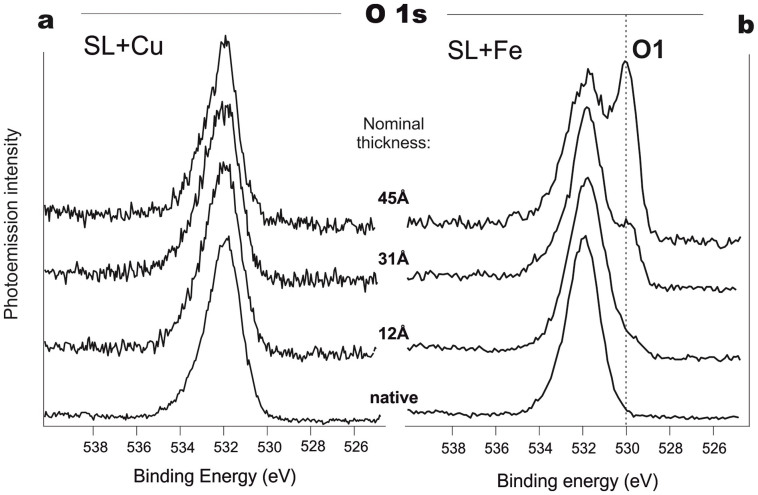
Core-level O 1s spectra taken for the native S-layer and after deposition of different thicknesses of copper (a) and iron (b) on top. The spectra were normalized each to its maximum intensity.

Upon further deposition of Cu (up to 45 Å), we observe a gradual growth of an *N2* component in the N 1s spectra. Once again, there is an absence of notable changes in O 1s (see Figure 2a) or C 1s core-level spectra (see Supplementary Information). In the case of Fe, when the total coverage reaches the 9–12 Å level, the appearance of a third feature, *N3*, at 397 eV BE becomes clearly visible (Figure 1b). Moreover, as Fe is further deposited this feature is continuously increasing; forming a well-defined peak that stands in contrast to the case of Cu. According to the literature data available for iron cyanide^25^ and iron nitrides^26^, the *N3* peak at 397 eV BE can indicate the formation of triple C≡N or Fe≡N bonds, with the first option being more probable for the system under consideration from the general chemical viewpoint.

The appearance of this *N3* feature in the PE N 1s spectra is not the only difference between Fe- and Cu-based systems. When analyzing the spectral pattern of the O 1s core-levels (Figures 2a and 2b) obtained by gradual deposition of Cu and Fe on the native SL samples, certain differences between them can also be seen. Clearly, for SL modified with Fe, we detect a new feature, *O1*, at 530 eV BE. This feature began to develop at a coverage of nearly 12 Å, and gradually forms a well-resolved peak when the deposited Fe reaches 45 Å. This peak could be related to metal oxide formation^27^,^28^,^29^. In contrast, only a weak shoulder is seen in this range of binding energies during Cu deposition.

To trace the chemical state of Fe and Cu in these hybrid systems we recorded the most informative spectra, which are anticipated to be X-ray photoemission spectroscopy (XPS) Fe 2p and near-edge X-ray absorption fine structure (NEXAFS) Cu L_3_.

**Figure 3:**
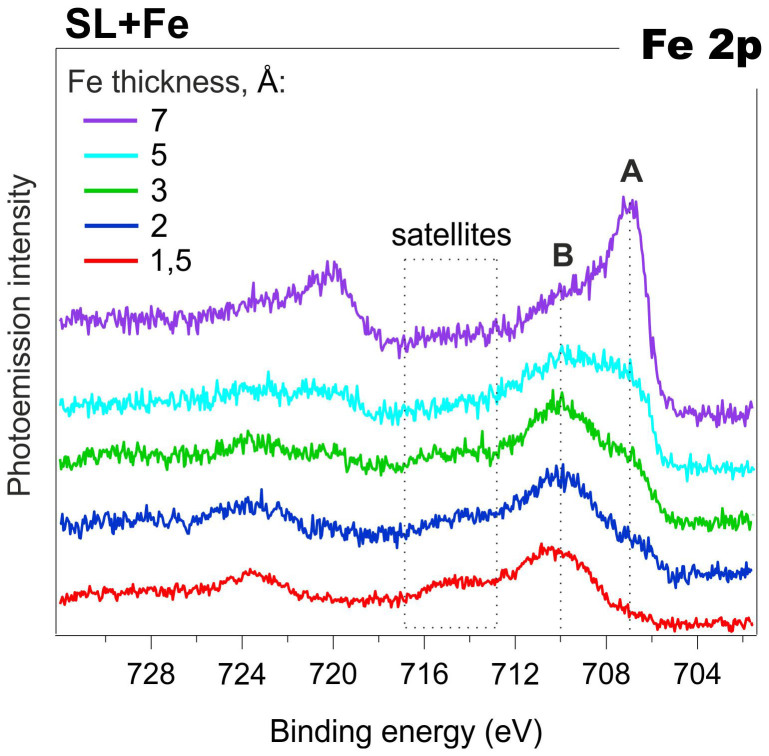
Core-level Fe 2p spectra taken for the small dose deposition of Fe on S-layer.

The evolution of the Fe 2p core-level photoemission lineshape is shown in Figure 3 as the metal coverage is increased to 7 Å. By analyzing the binding energy of features A and B, the possible iron charge states can be suggested by comparing them to the corresponding values in reference iron-based materials.

Feature *A* (at 707 eV), whose intensity increases with increasing thickness of the deposited metal, is apparently caused by the presence of elemental Fe^29^,^30^. It should be noted that the same BE is typical for the nanoparticles formed by iron atoms^31^,^32^. The broad component B at higher binding energies (>710 eV) most probably includes several peaks. This reflects multiplet splitting of the Fe 2p line, typically observed for the Fe^II^ (high-spin) and Fe^III^ species^33^. The presence of high-BE satellite features in the Fe 2p spectra indicates that iron is in the +2 and/or +3 state; i.e. iron oxidizes due to interaction with the protein. In order to identify the dominating iron state (Fe^II^ or Fe^III^), the relative position of the satellite features should be analyzed. It is well known that for Fe^II^, the satellite features are separated from the main peak by 4.3–6 eV. For Fe^III^ the corresponding peak BE difference is much larger, i.e. 8–8.5 eV^29^,^34^,^35^,^36^. In our case, in addition to the major component *B* at 710 eV, there are satellite features at BE 715 eV. This indicates that iron is most probably in the +2 state. When the metal coverage increases, the relative intensities of the satellites - as well as of the +2 state peak - decrease due to increasing contribution from metallic Fe. Therefore, at low coverage the component related to the oxidized iron atoms dominates; while above an overall thickness of 7–9 Å, the metallic iron component gives a major contribution to the spectra.

Since it is hard to distinguish between the Cu^I^ and Cu^0^ states based on only the XPS data^37^, we analyzed NEXAFS spectra taken for the L_3_-edge. As a reference sample for spectral features from copper nanoparticles, Cu was also deposited onto a freshly prepared graphite surface. The latter can be considered to be a model system for copper nanoparticles grown on an inert surface.

**Figure 4:**
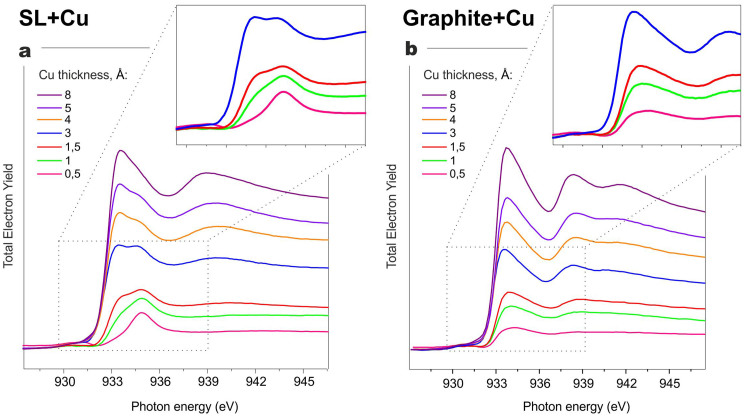
NEXAFS Cu L_3_-edge spectra for the S-layer covered by Cu (a) and the reference system which is graphite covered by the same amount of Cu (b).

A comparison of NEXAFS spectra taken at the Cu L_3_-edge for Cu gradually deposited on both an SL and on graphite is shown in Figures 4a and 4b, respectively. The fine structure of these spectra is caused by dipole allowed electron transitions from the 2p state to unoccupied electron states at the absorbing copper atom, with major contributions from the Cu 4s and Cu 3d states. The NEXAFS spectra of the reference system reflect the electronic structure of the nanoparticles, which differs from that of the bulk metal^38^,^39^. By comparing the NEXAFS spectra for both systems we can see, during the initial stage no metal particles are formed at the protein surface or, at least, their formation is a minor process. This supports the chemical nature of the interaction between protein and copper reflected by the N 1s XPS spectra.

Absence of a narrow, intensive pre-edge feature at 931 eV - a well-known indicator of the presence of Cu 3d^9^ state and a characteristic reference peak for the Cu^II^ compounds^37^,^40^ - confirms that there is no Cu^II^ in the SL + Cu system. Cu^I^ spectra reveal a wider, but less intense feature at higher photon energy. This reflects the 2p electrons' transition to certain unoccupied states of the compound. These are predominantly to Cu 4s, hybridized s,d-states with only a small d- admixture, or to free ligand orbitals. Such spectral features were also observed for the system under investigation at low metal coverage. As the metal coverage increases, the shape of the NEXAFS spectra alters to a three-peak pattern. This spectral shape reflects the unoccupied states of the bulk metal^37^,^41^. This indicates metal film or huge metal islands formation with a bulk-like chemical state and metallic properties. Indeed, the metallicity of deposited copper was also confirmed by appearance of the Fermi edge in the valence band spectra (not shown).

The corresponding spectra indicate that both Cu and Fe are rapidly oxidized during the initial stages of their deposition under vacuum onto native SL, leading to Cu^I^ and Fe^II^ states, respectively, while the process of metal nanoclusters and nanoparticles formation plays a minor role. At further deposition, the contribution of metal-like features to the spectral patterns notably increases suggesting efficient formation of various metallic nanostructures. Therefore, for chemically active metals like Cu and Fe, in addition to the chemical interaction at the interface that leads to bio-hybrid structure formation, we can also observe the growth of metal particles and islands.

On the other hand, the respective conclusion concerning cluster, particles and rather large metal islands formation can be drawn from the analysis of the intensity attenuation made for the core-level N 1s and O 1s spectra. It is a well-known fact that if the upper layer grows uniformly, i.e. in a layer by layer fashion, the intensity of PE lines of the substrate (SL in our case) constituents decreases exponentially with the increase of the layer thickness (see e.g. Ref. 42). In our experiments the corresponding dependence strongly deviated from the exponential equation. This also points out a lack of cover layer uniformity due to the formation of a variety of metal-based low dimensional structures (clusters, nanoparticles, islands) and, what is important, their coexistence with uncovered areas of the native S-layer (probably even at large amounts of deposited metal). In our experiments, therefore, we consider the term nominal thickness just as a measure of the amount of deposited metal.

**Figure 5:**
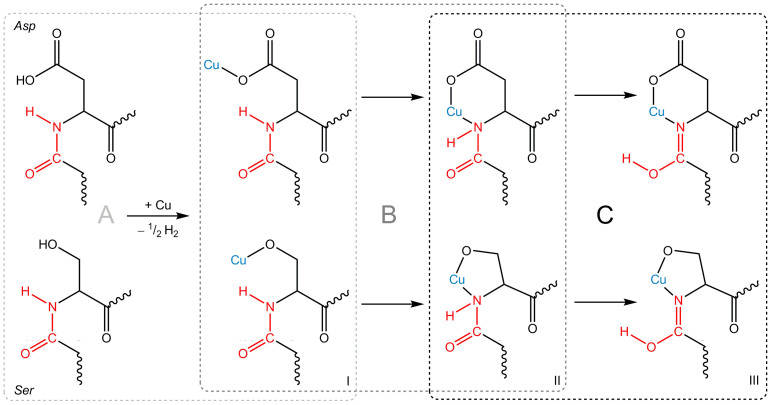
Schematic presentation of the redox process involving the carboxyl and hydroxyl functional groups of the protein (A) and the subsequent chemical bond reorganization (B, C) by the example of Aspartic acid and Serine side chains and copper.

The oxidation of metals is in line with general expectations, since the SL contains a sufficient quantity of terminal carboxyl and hydroxyl groups (in total amounting to 29% of the overall number of the functional groups^20^) to participate in the redox process. This process can be described as a metal oxidation and hydrogen reduction reaction, and is depicted in Figure 5A.

At the same time, the N 1s spectra (Figure 1) exhibit remarkable changes which arise from the formation of C = N bonds. This can be explained via the following proposed mechanism. Metal ions formed due to the reaction with protons (Figure 5A) are chelated via the formation of a metal-containing cycle^43^. Next, the equilibrium in the keto-enol tautomerism of the peptide bond (amide-iminol tautomerism) is shifted, as presented in Figure 5B, C. It should be noted that promotion of the keto-enol tautomerism upon incorporation of a metal ion into a polypeptide molecule has been reported earlier^44^. Further on, the –OH group of the enol form III (Figure 5C) can be involved in similar redox processes. Thus, the protein molecule becomes “armoured” by numerous covalent and donor-acceptor bonds between the metal ion and the protein.

Note that here we present a rather simple illustration of the elementary act of the metal-protein interaction in the case of copper. The situation with Fe is similar but, indeed, more complicated because iron has to be involved in two of these interactions to maintain its electrostatic balance.

**Figure 6:**
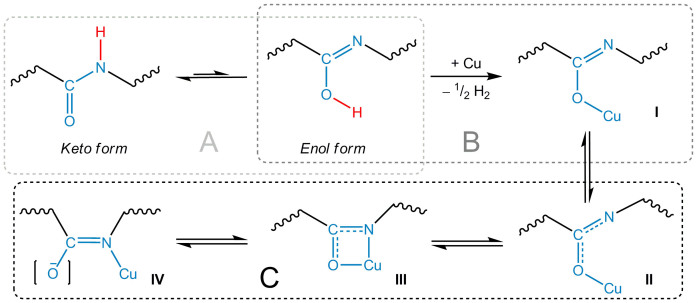
Keto-enol tautomerism of a peptide bond (A) followed by a redox process (B) and metal ion migration (C).

On the other hand, it is a well-known fact that peptide bonds in biomolecules undergo a process similar to the keto-enol tautomerism and do so in the absence of any external forces^45^ (Figure 6A). This means that peptide bonds in the protein molecule exist for a percentage of the time in the *enol* form, which features an –OH functional group. This group may also participate in the reaction that results in the metal oxidation (Figure 6B). A similar peptide bond deprotonation with a subsequent donor-acceptor bonding to a 3d metal ion has been previously reported^46^,^47^.

Further progression of the process presented in Figure 6A,B can give rise to a cation migration between the two donor centres (Figure 6B,C). It is clear that certain forms of the metal-protein structures (namely, form IV) must be stabilized additionally by another metal ion (or proton), which is incorporated into the protein “matrix”, probably due to a donor-acceptor interaction. The presence of isomers I–IV for different metals is restricted by their nature in terms of the Lewis acid/base classification^48^. Harder Fe^II^ ions will be predominantly coordinated by oxygen (Figure 6, I and II), while softer Cu^I^ will be coordinated by the imine bond/the Schiff base (Figure 6, IV). Note that both structures feature a multiple C−N bond. Form III is an intermediate.

It should be emphasized that the processes illustrated in Figures 5 and 6 are independent and can take place simultaneously. In the first process, the metal interacts with the side chain functional groups of the protein, which is followed by the peptide bond coordination to the metal ion, while the second one is a direct redox reaction of the metal involving the inner peptide bond.

All the processes described above take place with other donor protein groups and sites, thus “stitching” the metal cation into the protein network via both donor-acceptor and non-covalent interactions. For this reason, the proposed schemes (Figures 5 and 6) must be considered as a simplified model rather than a comprehensive process description.

The chemical processes illustrated in Figures 5–6 are in line with the observed changes in the XPS spectra for both Cu- and Fe-based SL systems during the initial stage of the interaction; namely, when a new component (*N2*) appears in the N 1s core-level spectra. The absence of any drastic changes in the spectra of other protein constituents is also in agreement with the proposed scenario, since hydrogen replacement by a metal atom in either carboxyl or hydroxyl groups does not cause any substantial chemical shift in the O 1s spectra^49^. The O 1s binding energy will also remain virtually unchanged during the peptide bond transformation and the *enol* structure stabilization^19^.

Further metal deposition causes different spectral changes for copper and iron. For the Cu-based system, no new features except for the hybrid formed at the interface are observed. This is presumably accompanied by copper cluster formation and metal film growth. Meanwhile, the Fe modified SL undergoes the next stage of metal-protein chemical interactions. The experimental data indicate that this step results in protein molecule destruction, with subsequent formation of iron oxides, carbides, cyanides or nitrides (Figures 1–2).

We anticipate that the difference between the copper and iron reactivity towards the SL can be interpreted as follows. During interaction with the protein, Fe reduces twice the number of protons as Cu. As a result, the protein looses twice –COOH and –OH functional groups for the same amount of coverage. In turn, the disappearance of these functional groups implies the destruction of the protein secondary and tertiary structures to a higher degree in the case of Fe, since these levels of structure are sensitive to the hydrogen bonds presence. In addition, the typical coordination numbers are 4 for Cu^I^ and 6 for Fe^II^; therefore, iron uses a proportionally larger amount of the protein donor groups than copper. Furthermore, formation of Fe inorganic species, including oxide^50^, seems to be more energetically (thermodynamically) favourable than for Cu; such iron species are tentatively more stable than the Fe-based SL hybrid structure. All these factors may contribute to the protein backbone degradation, which occurs during further metal-protein interaction for iron but not for copper. One should also take into account that the destruction of the secondary and tertiary protein structure due to partial loss of hydrogen and configuration changes in the protein-metal interaction in vacuum can cause an increased sensitivity to the X-ray impact which instigates chemical bond breaking, and thus the protein degradation in case of the Fe/SL system^51^. However, in our case this process seems to be of little importance compared to the chemical destruction of the protein as it interacts with iron.

In contrast, in the case of copper a stable protein-hybrid system is evidently formed, in which the initial primary structure is mostly conserved. Copper covers the SL surface, causing moderate chemical changes at the interface. Further increase the layer thickness does not seriously influence the Cu/SL interaction. This makes copper a potential candidate for possible future applications in metal-protein hybrid structures; and may find extensive use as, for example, electrical contacts, where stability of the metal/protein interface and the protein primary structure intactness should play an important role.

## Conclusions

Our study demonstrates for the first time that combined XPS and NEXAFS expertise can describe the chemical interaction between biomolecules like SL with technologically important elements such as Cu and Fe under vacuum conditions. An intrinsic feature of both hybrid systems is stabilization of the *enol* form of peptide bonds as the result of protein-metal interactions. However, the resulting hybrid with copper appears to be rather stable - in contrast to the system with iron, which is decomposed to form inorganic species like oxide, carbide, nitride, and cyanide. We have seen that at the early stage of deposition, metal oxidation to Cu^I^ and Fe^II^ is observed. These form a stable interface structure, especially in the case of copper. Furthermore, the stable metallic layer gradually grows on this structure. This observation demonstrates a potential application of Cu in development of novel bioinorganic hybrid systems under high vacuum conditions. In contrast to this, Fe reacts with protein more actively; and finally causes the degradation of the biomolecule.

## Methods

### Sample preparation

The SL was isolated from the bacterium *Lysinibacillus sphaericus* NCTC 9602. The cell cultivation has been described previously^10^. As sample substrates for the spectroscopic measurements we used naturally oxidized silicon wafers in 5 × 7 mm chips (TED PELLA, INC). The sample preparation was performed according to the protocols for the substrate cleaning as well as the SL deposition described elsewhere^52^. The morphology and coverage of the deposited SL were characterized by atomic force microscopy and transmission electron microscopy.

The hybrid metal-protein systems were synthesized in situ in the UHV by vapor deposition of high-purity metals (copper, iron) onto the surface of the SL immobilized at the silicon substrates. The metal deposition rate was calibrated using a quartz microbalance.

### Spectroscopic measurements

All spectroscopic measurements were performed at the Helmholtz-Zentrum Berlin für Materialien und Energie (HZB), the electron storage ring BESSY II, using the facilities of the Russian-German beamline^53^,^54^. This dipole-based beamline provides a moderate photon flux distributed continuously over a wide photon energy range from 30 to 1500 eV and, therefore, is particularly suited for radiation sensitive and fragile materials research^55^,^56^. XPS spectra were acquired with a hemispherical Phoibos 150 electron energy analyzer (Specs GmbH). The photoemission spectra were collected in similar kinetic energy ranges. To do so, photon energy of 520 eV, 650 eV, 850 eV were used for acquisition of the N 1s, O 1s, Fe 2p core-level spectra, respectively. For energy calibration all spectra were aligned relative to the Au 4f_7/2_ peak of a reference gold sample, set to 84.0 eV binding energy. NEXAFS spectra were recorded in a total-electron yield mode. All measurements were carried out at room temperature. The base pressure was 5 × 10^−10^ mbar and did not exceed 5 × 10^−9^ mbar during metal deposition. To check the X-ray protein damage effect, several points at the surface were studied for each sample. All positions of the photoemission and NEXAFS peaks are given in the manuscript with the ±0.1 eV accuracy.

## Author Contributions

All authors contributed to the design and/or execution of experiments and/or analyzed data. A.A.M., E.V.G., V.S.N. and L.V.Y. have performed XPS and NEXAFS experiments. A.B. and M.M. isolated the S-layer proteins, prepared the samples for the experiments and performed their TEM and AFM characterization. A.A.M., E.V.G., V.S.N., H.E. and D.V.V. wrote the manuscript. S.L.M., M.M., V.K.A., C.L. and D.V.V. have supervised the research. All authors have read and approved the decisive version of the manuscript.

## Supplementary Material

Supplementary Information Supplementary Information
