# Supplementary material for: Insight into Bio-metal Interface Formation in vacuo: Interplay of S-layer Protein with Copper and Iron
Source: Sci Rep. 2015 Mar 4;5:8710. doi: 10.1038/srep08710 (PMC4348631; doi:10.1038/srep08710)
Supplement: Supplementary Information [file srep08710-s1.pdf]

Supplementary Information

Insight into Bio-metal Interface Formation *in vacuo*: Interplay of S-layer Protein with Copper and Iron

Anna A. Makarova[1,2], Elena V. Grachova[3], Vera S. Neudachina[4], Lada V. Yashina[4], Anja Blüher[5], Serguei L. Molodtsov[6,7], Michael Mertig[5,8], Hermann Ehrlich[6], Vera K. Adamchuk[2], Clemens Laubschat[1], and Denis V. Vyalikh[1]*

[1] Institut für Festkörperphysik, Technische Universität Dresden, 01062 Dresden, Germany

[2] Department of Physics, St. Petersburg State University, 198504 St. Petersburg, Russia

[3] Department of Chemistry, St. Petersburg State University, 198504 St. Petersburg, Russia

[4] Department of Chemistry, Moscow State University, 119991 Moscow, Russia

[5] Professur für Physikalische Chemie, Mess- und Sensortechnik, Technische Universität Dresden, 01062 Dresden, Germany

[6] Institut für Experimentelle Physik, Technische Universität Bergakademie Freiberg, 09599 Freiberg, Germany

[7] European XFEL GmbH, 22761 Hamburg, Germany

[8] Kurt-Schwabe-Institut für Mess- und Sensortechnik e.V. Meinsberg, 04736 Waldheim, Germany

*Corresponding author: Denis.Vyalikh@tu-dresden.de

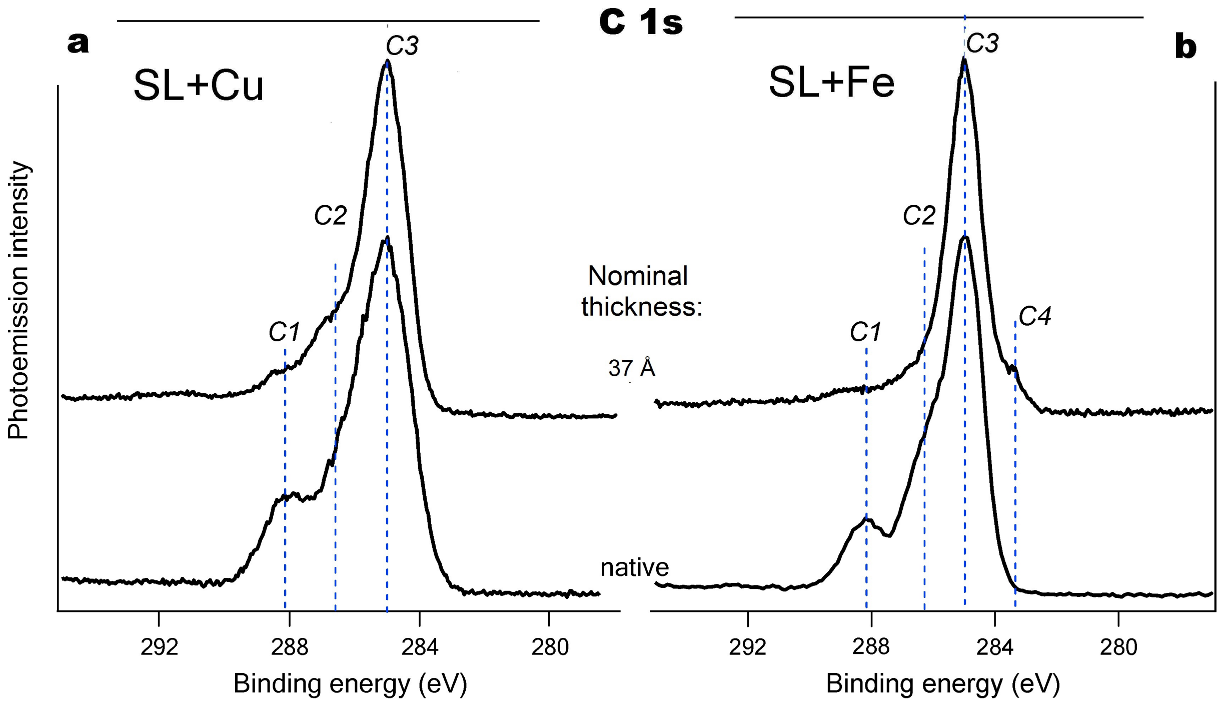

Supplementary Figure S1. Core-level C 1s spectra taken for the native S-layer and after deposition of 37 Å of copper (a) and iron (b) on top. The spectra were normalized each to its maximum intensity.

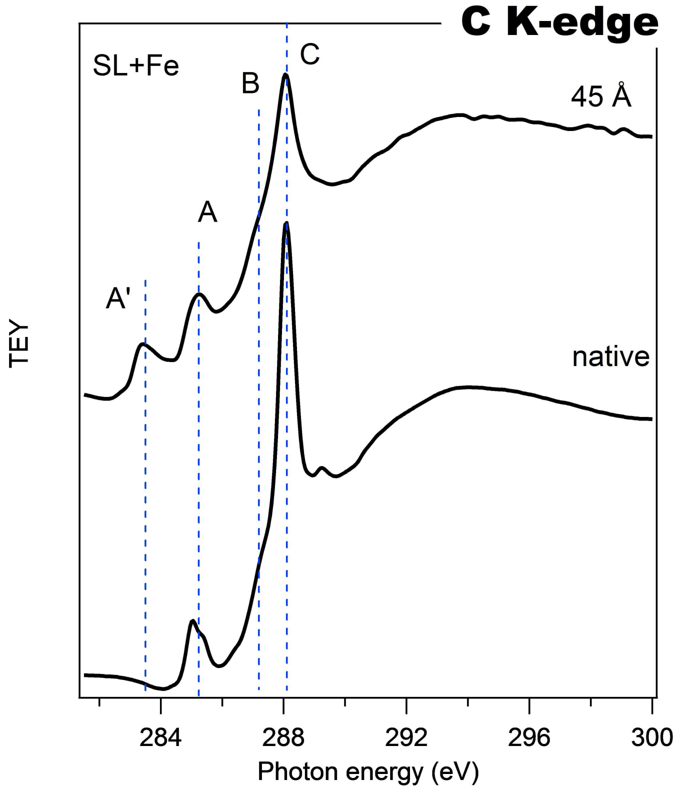

Supplementary Figure S2. C K-edge NEXAFS spectra taken for the native S-layer and after deposition of 45 Å of iron. The spectra were normalized to the edge jump.

Figure S1 shows C 1s core-level photoemission spectra taken for the native S-layer and after deposition of 37 Å of metals on top. In contrast to the simple shape of the O 1s and N 1s core-level photoemission spectra the structure of C 1s spectrum of the native S-layer protein is more complex reflecting a wide variety of chemical environments of carbon atoms in the protein. The spectrum for the native protein exhibits three distinct features where *C1* is due to carboxyl groups, *C2* is usually considered to be related to the amides/peptide bonds and hydroxyls, while *C3* is due to C-C and C-H bonds. Upon metal deposition the intensity of *C2* and especially *C1* peaks decreases relative to that of *C3.* It should be noted that for SL+Fe, in contrast to the SL+Cu system, we observe formation of a new feature *C4* with binding energy about 283.5 eV that indicates formation of iron carbides [S1] or cyanides [S2]. More detailed quantitative analysis of the C 1s spectra modifications, particularly at low coverages, is quite challenging task because carbon is also contained in the substrate and possibly contaminations because of the *ex situ* S-layer sample preparation.

As for the data obtained by NEXAFS at the absorption edges of the protein constituents (C, N and O K-edges), it is much less surface sensitive than the information acquired by XPS, and therefore less informative in our case of the predominately interface reaction. However, one important conclusion can be made on the basis of the C K-edge NEXAFS spectra, which are illustrated in Figure S2 for the native S-layer protein and after deposition of 45Å of iron: at large amounts of deposited iron a new spectral feature arises at ~283.5 eV photon energy (feature A’) that can be assigned to iron carbides [S3] and confirms our conclusions drawn from the C 1s core-level photoemission spectra interpretation.

Supplementary references

S1. Goretzki, H., v. Rosenstiel, P., Mandziej, S. Small area MXPS- and TEM-measurements on temper-embrittled 12% Cr steel. *Fresenius’ Zeitschrift für Analytische Chemie*, **333,** 451–452 (1989);

S2. Vannerberg, N. G. ESCA-spectra of sodium and potassium cyanide and of sodium and potassium salts of hexanometallates of 1st transition-metal series. *Chemica scripta.* **9**, 122–126 (1976);

S3. Gao, J. et al. Revealing the role of catalysts in carbon nanotubes and nanofibers by scanning transmission X-ray microscopy. *Sci. Rep.* **4**, 3606 (2014).
